# 25673 Training the Workforce During a Pandemic: Virtual Internships for Trainees

**DOI:** 10.1017/cts.2021.558

**Published:** 2021-03-30

**Authors:** Lauren Aleksunes, Yasheca Ebanks, Barbara Tafuto, Barbara Gladson, Doreen Lechner

**Affiliations:** Rutgers University

## Abstract

ABSTRACT IMPACT: Rapid launch of a virtual internship can address clinical and translational science training needs of the New Jersey Alliance for Clinical and Translational Science (NJ ACTS) workforce during a pandemic. OBJECTIVES/GOALS: The global pandemic has necessitated innovate strategies for training clinical and translational scientists. With trainees largely restricted from campus, the Workforce Development Core of the NJ ACTS Hub sought to develop and evaluate a virtual 8-week internship program for professional and graduate students within the NJ ACTS community. METHODS/STUDY POPULATION: Establishment of the internship required a systematic approach to 1) recruiting projects and supervisors, 2) collecting and evaluating 90 applications, 3) screening and selecting finalists, and 4) onboarding interns that spanned 7 weeks. Core Leads and Researchers within NJ ACTS developed 8 projects to be performed remotely by 11 interns. Leads and co-leads from the Team Science, Special Populations, Community Engagement, Informatics, and TL1 Cores and Programs designed projects. During the internship, participants engaged in a series of career development training and one-on-one mentoring through weekly meetings. The internship culminated in a final symposium open to the entire clinical and translational science community. RESULTS/ANTICIPATED RESULTS: Interns spanned different educational programs - pharmacy (36%), medicine (18%), and undergrad (9%)/graduate education (36%). Interns included women (63%), students from underrepresented backgrounds (27%) and students who were first in their family to pursue advanced education (18%). Project topics included competency assessment, COVID-19 clinical trials, marketing materials, community engagement salons, eSource for clinical trials, team science projects, and REDCap utilization. Using a 4-point Likert scale to evaluate competencies, the baseline strengths of interns included team-based science (mean/SD: 3.5 ±0.7). Trainings were designed to address gaps in intern skills including developing written and graphical abstracts (mean/SD: 2.2 ±0.9) and effective LinkedIn pages (mean/SD: 2.4 ±1.0). DISCUSSION/SIGNIFICANCE OF FINDINGS: Taken together, the rapid development and launch of a virtual internship program can increase participation of trainees in CTSA Hub research activities and address gaps in their clinical and translational skill set. Plans are to host virtual internships each semester to enhance workforce training and collaboration across Hub Cores.

